# Concomitant use of antidepressants and classic psychedelics: A scoping review

**DOI:** 10.1177/02698811251368360

**Published:** 2025-09-12

**Authors:** Stephan C. Tap, Kelan Thomas, Tomáš Páleníček, Dea S. Stenbæk, Albino J. Oliveira-Maia, Jens H. van Dalfsen, Robert A. Schoevers

**Affiliations:** 1 Department of Psychiatry, Groningen University Medical Centre, Groningen, the Netherlands; 2 College of Pharmacy, Touro University California, Vallejo, CA, USA; 3 Psychedelic Research Centre, National Institute of Mental Health, Klecany, Czech Republic; 4 Department of Psychology, University of Copenhagen, Denmark; 5 Neurobiology Research Unit, Copenhagen University Hospital, Denmark; 6 Champalimaud Research and Clinical Centre, Champalimaud Foundation, Lisbon, Portugal. NOVA Medical School, Faculdade de Ciências Médicas, NMS, FCM, Universidade NOVA de Lisboa, Lisbon, Portugal

**Keywords:** classic psychedelics, antidepressants, safety, treatment efficacy, subjective effects

## Abstract

Classic psychedelics are increasingly studied as potential treatments for different psychiatric disorders. Current research protocols often require patients to discontinue antidepressants (ADs) for at least 2 weeks before psychedelic administration to decrease the risk of serotonin syndrome and limit their effect on efficacy and the acute subjective effects of psychedelics. Moreover, the discontinuation of ADs represents a significant burden to patients that could also worsen their depression status and increase suicidal ideation. Together, this suggests that the general recommendation for AD discontinuation might be unnecessary and even detrimental to the therapeutic efficacy of psychedelics. In this scoping review, we summarise the existing literature on the concomitant use of conventional ADs with classic psychedelics in humans with the aims to assess safety, tolerability, efficacy, and subjective effects. Following PRISMA-ScR guidelines, we searched MEDLINE, Embase, and Scopus databases to retrieve relevant literature from inception to March 3, 2025. Data were systematically charted from included studies. We included 18 studies and found that the concomitant use of ADs and classic psychedelics is generally safe and tolerable, with no increased risk of serotonin syndrome, particularly for psilocybin. Some studies reported significant improvements in depression and other mental health symptoms. While some evidence indicates a potential attenuation of acute subjective psychedelic effects, this was not observed in all studies. Accordingly, we conclude that the use of ADs can be maintained to enhance patient access to psychedelic treatments and avoid the risk of AD discontinuation syndrome. Finally, this review highlights limitations and several knowledge gaps in the current literature that need to be addressed in future randomized double-blind, placebo-controlled trials.

## Introduction

In the past decade, interest in the therapeutic use and potential of classic psychedelics in the treatment of various psychiatric disorders has rapidly increased (Hadar et al., 2022). Recent reviews have supported the promising therapeutic efficacy of psychedelics, often combined with psychological support (Johnson et al., 2008), with rapid and sustained improvements in a sizeable proportion of patients with major depressive disorder (MDD) (Andersen et al., 2021; Perez et al., 2023).

In real-world environments, symptoms of MDD are typically managed with various monoaminergic antidepressants (ADs), which include selective serotonin reuptake inhibitors (SSRIs), serotonin and noradrenaline reuptake inhibitors (SNRIs), noradrenalin and dopamine reuptake inhibitors (NDRI), tricyclic ADs (TCAs), monoamine oxidase inhibitors (MAOIs), and atypical ADs (e.g. bupropion, mirtazapine, vortioxetine) (Dold et al., 2016; Kim et al., 2021; Luo et al., 2020). Most of these conventional ADs seek to increase intrasynaptic levels of serotonin, which is thought to be the mechanism leading to a reduction of depressive symptoms (Moncrieff et al., 2023), either through inhibiting the reuptake of serotonin (i.e. SSRIs) and other neurotransmitters (i.e. SNRIs, NDRIs, TCAs), or by blocking the enzyme monoamine oxidase (i.e. MAOIs) that in turn prevents the breakdown of serotonin in the synaptic cleft (Belmaker and Agam, 2008).

Classic psychedelics also affect serotonin neurotransmission, primarily through agonism of post-synaptic 5-HT_2A_ receptors (Nichols, 2016). Essentially, on a theoretical basis, it has been assumed that the concomitant use of ADs with classic psychedelics could synergistically increase serotonin neurotransmission and potentially cause serotonin toxicity (Malcolm and Thomas, 2022), which refers to a drug-induced “toxidrome” in which intrasynaptic serotonin levels are elevated as a result of drug-drug interactions (Gillman, 2006). This excess intrasynaptic serotonin agonism is responsible for serotonin syndrome that manifests with a range of clinical symptoms, such as tremor and diarrhea in mild cases, or delirium, neuromuscular rigidity, seizures, coma, and hyperthermia in more severe cases (Boyer and Shannon, 2005). Furthermore, early studies indicate that the concomitant use of ADs decreases the acute subjective effects of psychedelics (Bonson, 1996; Grof and Dytrych, 1965; Strassman, 1992), which are assumed to be responsible for their enduring therapeutic effects (Hovmand et al., 2023; Yaden and Griffiths, 2021). Accordingly, to avoid the risk of serotonin toxicity and fully isolate the antidepressant effect of serotonergic psychedelics, patients enrolled in most contemporary clinical trials are typically required to discontinue ADs at least 2 weeks before drug administration (Carhart-Harris et al., 2021; Davis et al., 2021; Goodwin et al., 2022; Raison et al., 2023; Sloshower et al., 2023; von Rotz et al., 2023).

However, the discontinuation of ADs takes time and represents a significant burden for patients, as it may lead to worsening of depressive symptomatology and increased suicidal ideation that would then occur prior to a potentially challenging psychedelic experience, either through rebound effects, AD discontinuation syndrome, or by interrupting a (partially) effective treatment (Davies and Read, 2019; Kato et al., 2021; Lewis et al., 2021). Importantly, the idea of increased risk for developing serotonin syndrome and/or serotonin toxicity, when ADs are co-administered with high doses of psychedelics, has recently been challenged, in part because classic psychedelics are partial agonists of the 5-HT_2A_ receptor and would also compete for serotonin binding (Malcolm and Thomas, 2022; Rickli et al., 2016; Sarparast et al., 2022). Based on this clinical rationale, the concomitant use of ADs and classic psychedelics may be preferred, or patients could temporarily reduce the dose of ADs around dosing days with psychedelics to have minimal interactions. Simultaneously, the competition for 5-HT_2A_ receptors could impede the biological action of psychedelics during concomitant use of ADs and potentially limit efficacy (Halman et al., 2024), particularly as the 5-HT_2A_ receptor induces neuroplasticity (Cameron et al., 2023; Ly et al., 2018; Vargas et al., 2023).

In this scoping review, we aim to identify sources of evidence and gaps in existing research on the concomitant use of ADs and psychedelics in humans (Amog et al., 2022; Munn et al., 2018). The main objective is to obtain information on safety and whether the concomitant use of ADs significantly increases serotonin toxicity risk during psychedelic treatment. We also explored the potential impact of ADs on psychedelic treatment efficacy and acute subjective effects.

## Methods

We conducted a scoping review to summarize the existing literature on the concomitant use of conventional ADs and classic psychedelics. Because the extent of existing literature on this topic was initially unclear and we aimed to identify and analyse knowledge gaps (Munn et al., 2018), our research team determined that a scoping review was the most appropriate methodology. This decision was further supported by the web-based “Right Review” tool (Amog et al., 2022), which serves as a decision support system to confirm methodological suitability. Accordingly, we employed the methodological framework outlined by Arksey and O’Malley (Arksey and O’Malley, 2005) and followed the most recent JBI guidelines for conducting scoping reviews (Peters et al., 2020).

A comprehensive search of electronic databases was performed in MEDLINE (PubMed), EMBASE, and Scopus to retrieve relevant literature investigating the concomitant use of conventional ADs and classic psychedelics. The search string included terms relevant to “psychedelics,” “antidepressants,” and “concomitant use” (see Appendix A for the complete search strategy). The systematic search followed PRISMA-ScR guidelines (Tricco et al., 2018) and was conducted from inception until June 27, 2025.

Articles to be considered for this scoping review needed to focus on the concomitant use of conventional ADs and classic psychedelics. Peer-reviewed journal papers were included if they involved human participants. Quantitative, qualitative, and mixed-method studies were included in order to consider different aspects of the concomitant use of conventional ADs and classic psychedelics and their impact on safety, treatment efficacy, and acute subjective effects. There were no restrictions on language or publication date. Articles identified in all three databases were screened for eligibility by SCT. Reference lists of eligible studies were reviewed to identify additional studies (Tricco et al., 2018). Articles that did not fit the research question of this scoping review or with irrelevant titles and abstracts were excluded, as were studies involving animals.

**Figure 1.**
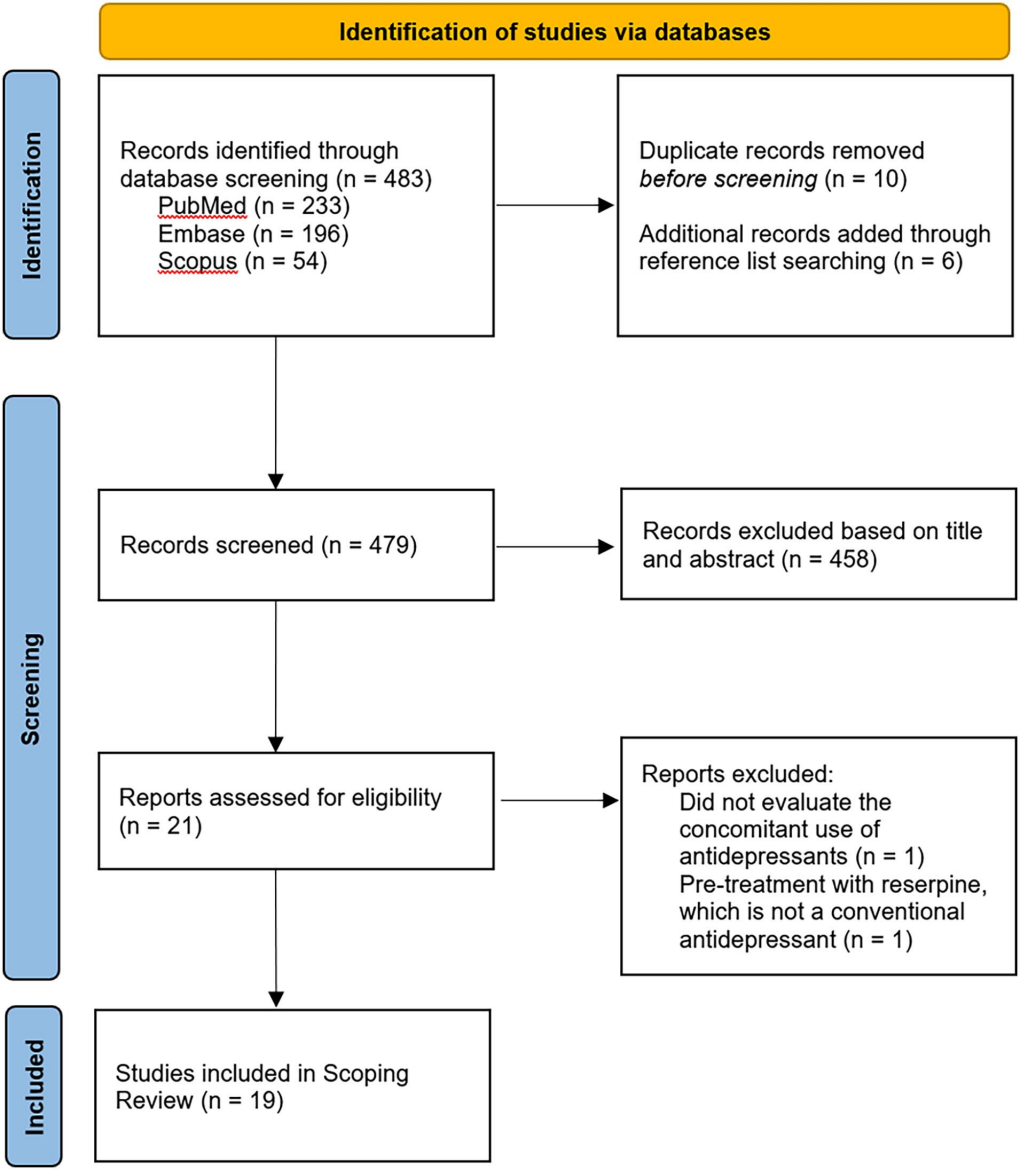
Screening and selection of articles

Data were extracted according to a list of parameters, including the last name of the first author, year of publication, study design (e.g. randomized clinical trial, open-label, observational, case study), study population and sample size, psychedelic substance (e.g. psilocybin), dose (milligrams, micrograms, or millilitres), number of doses, psychological support, setting (e.g. environment setup), class of AD, dose of AD (if available), length of AD treatment, other concomitant psychotropic medications, safety in terms of serotonin syndrome and related (serious) adverse events, treatment efficacy, and acute subjective effects. Considering the infancy of this particular topic and limited number of studies (see Figure 1 and Table 1), only SCT as first author of this article extracted data from articles and supplementary materials. All co-authors of the article reviewed the manuscript for correct interpretation of the data and provided input where necessary.

**Table 1.** Overview of studies involving the concomitant use of conventional antidepressants and classic psychedelics.

Reference	Study design	Study population	Psychedelic	Psychological support	Setting	Antidepressant	Other psychotropic medications	Safety	Treatment efficacy	Subjective effects
Falchi-Carvalho et al. (2025)	Open-label trial	TRD (*n* = 14)	DMT (15 mg and 60 mg)	One preparation session (n.s.) to inform about DMT’s effects, establish rapport, manage expectations, and provide strategies for challenging experiences. Two dosing sessions (1 h and 2 h) Two immediate post-dosing integration sessions (30 min and 60 min) to report experience and draw mandala, respectively. Two online integration sessions, 1 day (n.s.) and 7 days (n.s.) after DMT to facilitate meaning-making, address emotional or cognitive challenges from the acute phase, and promote insights to sustain therapeutic benefit	Modern living room style featuring warm colours, paintings, dimmed lighting, smoothly changing LED lights, and medical devices. Patients lay on recliner with eyeshades and listened to music.	P1: SNRI venlafaxine (150 mg) P2: SNRI desvenlafaxine (50 mg) P4: SNRI desvenlafaxine (150 mg) P6: SSRI escitalopram (10 mg) P7: atypical AD bupropion (300 mg) and SARI trazodone (100 mg) P8: SMS vortioxetine (10 mg) P9: SSRI escitalopram (20 mg) and SARI trazodone (50 mg) P10: SSRI citalopram (20 mg) P11: SNRI desvenlafaxine (200 mg) and atypical AD bupropion (300 mg) P14: atypical AD bupropion (450 mg) and SMS vortioxetine (5 mg)	P1: zolpidem (10 mg) P2: none P4: aripiprazole (5 mg) and sodium valproate (1000 mg) P6: pregabalin (150 mg) and lithium (300 mg) P7: clonazepam (2 mg) P8: lisdexamfetamine (50 mg), zolpidem (10 mg), and pregabalin (75 mg) P9: aripiprazole (5 mg) P10: pregabalin (50 mg) P11: none P14: eszopiclone (3 mg)	Acute AEs were mild and transient, including pharyngeal discomfort (*n* = 16), cough (*n* = 12), and headache (*n* = 9). DMT increased systolic blood pressure (*p* < 0.02), diastolic blood pressure (*p* < 0.001), and heart rate (*p* < 0.001). No significant increases were observed for oxygen saturation (SpO2) (*p* = 0.08) and respiratory rate (*p* = 0.52). There were no significant differences between the AD and non-AD group in terms of safety.	Decreases in MADRS scores were marginally significantly larger in the AD group (*U* = 7.5; *p* = 0.07). The response and remission rates 1 week after DMT were 100% and 70% for the AD group and 50% and 25% for the non-AD group, respectively.	No significant difference in the intensity of the psychedelic experience as measured by VAS (*p* = 0.98) between AD group (*M* = 86.35, SD = 15.85) and non-AD group (*M* = 79.42, SD = 34.66)
Becker et al. (2025)	Randomized, double-blind, placebo-controlled, crossover study	Healthy volunteers (*n* = 23)	LSD (100 mcg)	NA	“Calm” hospital room	SSRI: 7 days of paroxetine (10 mg) followed by 35 days of paroxetine (20 mg) versus 42 days of placebo (mannitol) pre-treatment	NA	Paroxetine significantly reduced LSD-induced elevations in heart rate compared with placebo (*p* = 0.025), but did not affect QTc interval, blood pressure, mean arterial pressure, rate pressure product, or body temperature (*p* > 0.05) AEs were similar for acute LSD effects (0-12h) in placebo (*M* = 7.9, SD = 6.6) and paroxetine group (*M* = 6.8, SD = 7.8) and for subacute LSD effects (12-14h) in placebo (*M* = 2.3, SD = 4.5) and paroxetine group (*M* = 1.1, SD = 3.2). The most frequently reported AE was headache (placebo: *n* = 13, paroxetine: *n* = 16), followed by nausea (placebo: *n* = 13, paroxetine: *n* = 6). No severe AEs occurred during the study.	NA	The duration of LSD (in hours) was similar after paroxetine (*M* = 9.1, SD = 2.3) and placebo (*M* = 9.6, SD = 2.4). Paroxetine did not significantly influence VAS ratings of “drug high,” “feeling depressed,” “visual” and “auditory alterations,” “synaesthesia,” “altered perception of time,” “ego dissolution,” “insight,” “talkative,” “open,” “trust,” or “personal thoughts,” compared with placebo. Paroxetine did not significantly influence ratings on the Psychedelic Experience Scale, Adjective Mood Rating Scale, or 5D-ASC dimensions compared with placebo Paroxetine significantly reduced VAS ratings of “bad drug effect” (*p* = 0.04) and “anxiety” (*p* = 0.04).
Barnett et al. (2024)	Case study	TRD (*n* = 1)	*First dose* Dried batch of unknown *psilocybe* mushrooms (3 g) *Second dose* Dried batch of *psilocybe cubensis* mushrooms (1 g)	NA	Uncontrolled, naturalistic setting	*First dose* MAOI: phenelzine (45 mg twice daily) TCA: nortriptyline (100 mg nightly) *Second dose* MAOI: 2 weeks of tranylcypromine (20 mg twice daily)*	*First dose* Propranolol (20 mg three times daily), valproate extended release (2000 mg nightly), zolpidem (10 mg nightly), and lithium (20 mg twice daily) *Second dose* Extended-release dextroamphetamine-amphetamine (20 mg daily)	*First dose* No (serious) AEs *Second dose* Patient appeared to be “distressed.” Severe hypertension (200/160 mmHg and 220/140 mmHg), 10/10 (10 being most severe) intense chest pain radiating to the jaw, heart palpitations, severe headache, nausea, and abdominal pain. Diagnosis of hypertensive emergency and acute coronary syndrome. Diagnosis of ST-elevation myocardial infarction (STEMI) characterized by sinus brady-cardia, QT prolongation, and AVR elevation with lateral depressions.	*First dose* Significant improvement in mental health (“the best [he has] ever had” *Second dose* NA	*First dose* Complete absence of acute subjective effects. *Second dose* Complete absence of acute subjective effects.
Sakai et al. (2024)	Qualitative	General population (*n* = 443)	Psilocybin (NA)	NA	Uncontrolled, naturalistic setting	SSRIs: escitalopram (NA) or sertraline (NA) was mentioned in over 50% of posts		Two posts were suggestive of severe serotonin toxicity (e.g. seizure and muscle rigidity) 8% of posts (*n* = 37) reported negative psychological (e.g. paranoia, memory loss, or confusion) and physical effects (e.g. headache, nausea, and vomiting).	NA	54% (*n* = 238) of posts reported a decrease in intensity of psilocybin effects. 39% (*n* = 175) of posts reported no change in intensity of psilocybin effects. 1% (*n* = 5) of posts reported increased intensity.
Do et al. (2024)	Case study	DTD (*n* = 1)	*First dose* Psilocybin (25 mg) *Second dose* Psilocybin (25 mg) (7 weeks later)	“Psilocybin-assisted psychotherapy” (n.s.)	NA	*First dose* SNRI: duloxetine (60 mg daily) Atypical AD: vortioxetine (20 mg daily) *Second dose* Atypical AD: vortioxetine (20 mg daily)	*First dose* Zolpidem (10-15 mg) and quetiapine (25-50 mg)† *Second dose* n.s.	*First dose* Mild headache and mild increases in heart rate (63 to 66) and blood pressure (119/77 to 141/93 mm Hg) at 90 minutes post-psilocybin ingestion. *Second dose* Psilocybin was well-tolerated with no significant increases in heart rate (59 to 61) and blood pressure (128/87 to 134/91 mm Hg).	*First dose* Improvements in depression, anxiety, and suicidality 1 week after the first dose of psilocybin. *Second dose* Worsening of symptoms in depression, anxiety, and suicidality 1 week after second dose of psilocybin‡.	*First dose* Emotional Breakthrough Inventory score = 60§ *Second dose* NA
Barbut Siva et al. (2024)	Prospective survey	Self-reported psychiatric diagnosis (*n* = 131)¶	Psilocybin and LSD|| (NA)	NA	Uncontrolled, naturalistic setting	SSRIs and SNRIs (NA)	NA	NA	Significant improvements in symptoms of well-being (*p* < 0.001) and depression (*p* < 0.001) at 4-week follow-up in individuals with and without Ads.	Significant decreases in Mystical (*p* = 0.048), Challenging (*p* = 0.001), and Emotional Breakthrough Experiences. (*p* = 0.02) in individuals with ADs compared to participants without ADs.
Gukasyan et al. (2023)	Retrospective survey	General population (*n* = 611)	Psilocybin (NA)	NA	Uncontrolled, naturalistic setting	< 1 month and > 12 months of SSRIs, SNRIs, and/or atypical AD (bupropion) (NA)	NA	4% indicated an AE (unspecified) and 2.8% believed they had developed serotonin syndrome after reading a provided description. No reports of hospital visits or diagnoses.	NA	549 (51.7%) reported reduced intensity. 313 (29.5%) reported the same intensity. 128 (12.1%) reported they were not sure. 72 (6.8%) reported more intensity.
Goodwin et al. (2023)	Open-label trial	TRD (*n* = 19)	Psilocybin (25 mg)	Three preparation sessions (n.s.) with one trained mental health professional to build trust, explain trial procedures, and provide psychoeducation. One dosing session (6–8 h) with one lead and one assistant therapist for safety and to encourage internal focus on the experience. Two integration sessions (n.s.) with lead therapist 2 days and 1 week after psilocybin, to derive solutions and insights from psilocybin.	Patients lay on a couch in a room with dim lights while listening to a standardized playlist with relaxing music, along with continuous supervision and support from a trained therapist.	SSRIs: average use of 14.68 months of sertraline, escitalopram, fluoxetine, vilazodone, paroxetine, and citalopram.	NA	Treatment-emergent AEs included transient mild to moderate headaches (*n* = 6) that resolved spontaneously or were treated with paracetamol and ibuprofen. Other treatment-emergent AEs included increased blood pressure (*n* = 3) and were treated with clonidine. One patient experienced chest heaviness and headache and received clonidine. No increases in suicidal ideation and no clinically meaningful changes in clinical laboratory test, ECG, and QTc interval from baseline to follow-up (3-weeks). All treatment-emergent AEs lasted no longer than 7 days	Improvement in depression severity from Baseline to Week 3 (mean change MADRS total score: −14.9 (95% CI, −20.7 to−9.2). Remission rates (MADRS ⩽10) were 52.6% at Day 2, 47.4% at Week 1 and 42.1% at Week 2, and 42.1% at Week 3. Improvement in illness severity from Baseline to Week 3 (mean change CGI-S total score: −1.3 (SD = 1.29).	Acute subjective effects observed in terms of 5D-ASC “Oceanic Boundlessness” (*M* = 47.32, SD = 30.38), “Anxious Ego Dissolution” (*M* = 24.17, SD = 20.27), “Visual Restructuralization” (*M* = 51.72, SD = 27.19), “Auditory Alterations” (*M* = 17.43, SD = 20.72), and “Reduction of Vigilance” (*M* = 43.70, SD = 24.36).
Rosenblat et al. (2023)	Case study	TRD (*n* = 1)	Psilocybin (25 mg)	One preparation session (1–2 h) with two therapists to provide information about psilocybin’s effects, build rapport, and setting intentions. One dosing session (6–8 h) with two therapists for safety, support, and to encourage internal focus. Two integration sessions (1–2 h each) with two therapists to reflect on previously set intentions and facilitate meaning-making.	Patients lay on a couch while listening to a pre-selected music playlist in a “clinical” setting with two trained therapists.	SARI: trazodone (200 mg nightly)	NA	NA	Rapid, robust, and sustained antidepressant effects with full remission of depression. MADRS score decreased from 30 at baseline to 15 one day post-psilocybin. MADRS score ranged from 7 to 12 throughout the 6-month observation period	Complete absence of acute subjective effects.
Becker et al. (2022)	Randomized, double-blind, placebo-controlled, crossover study	Healthy volunteers (*n* = 23)	Psilocybin (25 mg)	NA	“Calm” hospital room	SSRI: 7 days of escitalopram (10 mg) followed by 7 days of escitalopram (20 mg) versus 2 weeks of placebo (mannitol) pre-treatment.	NA	Escitalopram significantly decreased peak systolic blood pressure (*p* < 0.001), peak diastolic blood pressure (*p* = 0.017), rate pressure product (*p* = 0.001), mean arterial pressure (*p* = 0.002), and pupil dilation (*p* = 0.002) compared to placebo. Escitalopram significantly reduced acute adverse effects, assessed by List of Complaints (*p* = 0.028), compared to placebo. Escitalopram was not associated with different or more frequent AEs after psilocybin compared to placebo and included headaches (*n* = 6 in escitalopram, *n* = 6 in placebo), flashbacks (*n* = 1 in escitalopram, *n* = 1 in placebo), nausea (*n* = 1 in escitalopram, *n* = 1 in placebo), abdominal bloating (*n* = 1 in escitalopram), vasovagal syncope (*n* = 1 in placebo), and lack of energy (*n* = 1 in escitalopram). Escitalopram did not significantly alter QTc interval before or after psilocybin compared to placebo (*p* > 0.05).	NA	Significant decreases in 5D-ASC “Anxious Ego Dissolution” (*p* = 0.029), 5D-ASC “Anxiety” (*p* = 0.026) compared to placebo. Other 5D-ASC dimensions were increased and not significantly different compared to placebo (*p* > 0.05). Significant decreases in MEQ-43 “Nadir Effects” (*p* = 0.001) and MEQ-30 “Ineffability” (*p* = 0.02) compared to placebo. Other MEQ dimensions were not affected (*p* > 0.05). Significant decreases in self-report ratings of “any drug effects” (*p* = 0.015), “bad drug effects” (*p* = 0.004), “fear” (*p* = 0.004), “talkative” (*p* = 0.029), “open” (*p* = 0.027), “happy” (*p* = 0.041), “concentration” (*p* = 0.012), “anxiety” (*p* = 0.007) compared to placebo.
Callaway & Grob (1998)	Case report	Mild MDD (*n* = 1)	*Ayahuasca* (100 ml)	NA	Uncontrolled, naturalistic setting	SSRI: several months (unspecified) of fluoxetine (20 mg)	NA	Tremors, sweating, shivering, and confusion (1h after ayahuasca). Subject continued to sweat profusely, display gross motor tremors and experience severe nausea and vomiting for 3 h along with disorientation. Subject became asymptomatic without treatment (4h after ayahuasca). There were no long-term adverse sequelae.	Experience with ayahuasca was considered valuable in helping him and his wife reconcile after being unfaithful to her.	Profound despair and anguish associated with mental imagery of subject’s wife and the “horrible pain” she experienced because of his infidelity.
Bonson (1996)	Qualitative study	MDD (*n* = 28), anxiety (*n* = 1), dysthymia (*n* = 1), PTSD (*n* = 1), ADD (*n* = 1), and stress (*n* = 1)	LSD (estimated doses ranging from 75 mcg to 500 mcg and from “moderate” to “very high”)	NA	Uncontrolled, naturalistic setting	SSRIs: ~3 and 150+ weeks of fluoxetine, sertraline, and paroxetine SARI: ~3 and 150+ weeks of trazodone	NA	NA	NA	Subjective decrease or “virtual elimination” of response to LSD. One subject who took the SSRI fluoxetine for 1 week had an increased subjective response to LSD.
Bonson & Murphy (1995)	Qualitative study	MDD (*n* = 10)	LSD (NA)	NA	Uncontrolled, naturalistic setting	MAOIs: ~3 and 150+ weeks of phenelzine and/or tranylcypromine TCAs: ~3 and 150+ weeks imipramine, desipramine, clomipramine	NA	NA	NA	MAOIs were associated with decreases in subjective LSD response. TCAs were associated with increases in subjective LSD response.
Strassman (1992)	Case study	Obsessionality and dysthymia (*n* = 1)	LSD (NA)	NA	NA	SSRI: fluoxetine (20 mg)	NA	NA	NA	Significant decrease in subjective effects with approximately one-half to one-third of producing the “desired effects.”
Vojtěchovský et al. (1968)	Placebo- controlled trial	Abstinent alcoholic patients (*n* = 9)	Psilocybin (20 mg)	NA	NA	MAOI: 2 days of tranylcypromine (80 mg) versus 2 days of placebo (NA)	NA	Premedication of tranylcypromine potentiated increases in systolic blood pressure (no data reported), diastolic blood pressure, mydriasis, while preventing the decline in emotional dynamogeny and mental alertness compared to placebo. AEs consisted of insomnia, dry mouth, and thirst in both groups.	NA	Premedication with tranylcypromine did not significantly reduce the acute subjective effects of psilocybin.
Grof & Dytrych (1965)	Open-label trial	Neurotic patients (*n* = 11) Neurotic patients (*n* = 3)	LSD (up to 400 mcg) LSD (150 mcg up to 500 mcg)	NA	NA	MAOI: Several weeks (unspecified) of nialamide (250–500 mg daily and shifted after several days to 150–300 mg daily) followed by a 24-h washout. MAOI: 3 weeks of daily nialamide (reaching a total amount of 3500 mg) followed by a 24-h washout (unspecified).	NA	NA LSD 150 mcg: reports of “massive psychotic symptomatology.” (unspecified). LSD 500 mcg: minimal subjective complaints.	NA NA NA	Minimal or no reaction to LSD. A “strong resistance” against LSD lasting at least 14 days. NA NA
Resnick et al. (1964)	Open-label trial	Healthy volunteers (*n* = 4)	LSD (40 mcg) LSD (40 mcg) LSD (40 mcg) LSD (75 mcg) LSD (75 mcg) LSD (75 mcg)	NA	NA	No AD MAOI: 2 weeks of isocarboxazide (30 mg daily) MAOI: 5 weeks of isocarboxazide (30 mg daily) No AD MAOI: 2 weeks of isocarboxazide (30 mg daily) MAOI: 5 weeks of isocarboxazide (30 mg daily)	NA	Pretreatment with isocarboxazide significantly diminished (or even reversed) the psychological (Apparent Eye-Level Test and Stroop Color-Word Test – Card C), autonomic (systolic and diastolic blood pressure, heart rate, and pupillary size), and neurological effects (sensory, motor, and autonomic systems, cerebellar functions, and the deep tendon and periosteal reflexes) typically produced by LSD-25**. All assessments were carried out before LSD and at 0.5, 1, 2, 3, 4, and 5 h after LSD.	NA	Complete absence or significant decreases in acute subjective effects (confirmed by a symptom rating scale, and by interview and behavioral observations carried out by three investigator). All four subjects stated that their experience with LSD-25 following pretreatment with isocarboxazide differed significantly from their experience without isocarboxazide pretreatment.
Sai-Halaz (1963)	Open-label trial	Healthy volunteers (*n* = 7)	*Reduced dose* DMT (0.35–0.55 mg/kg, *n* = 2) *Higher dose* DMT (0.65–0.83 mg/kg, *n* = 5)	NA	NA	*Reduced dose* MAOI: 4 days of iproniazid (100 mg) pretreatment followed by 48-h washout period *Higher dose* MAOI: 4 days of iproniazid (100 mg) pretreatment followed by 48-h washout period	NA	*Reduced dose* NA *Higher dose* NA	*Reduced dose* NA *Higher dose* NA	*Reduced dose* No hallucinations, no disturbances in time and space orientation. Odd feeling, not completely normal, and the outside world had something “strange” in it. *Higher dose* In some individuals, illusions and hallucinations occur without colors (only with eyes closed). Hallucinations were agitated and sense of time was lost. Everything was normal with eyes open. After 14–24 min, hallucinations disappeared and time-orientation came back. The feeling of not being completely normal remained and increased.
DeMaar et al. (1960)	Double-blind placebo-controlled trial	Healthy volunteers (*n* = 16)	LSD (25, 50, or 100 mcg)	NA	Hospital room	MAOI: iproniazid (100 mg) versus placebo (reserpine) (2 mg)	NA	Premedication with iproniazid resulted in attenuated pupillary dilation and diminished changes in both diastolic and systolic blood pressure (unspecified)***.	NA	No significant changes in acute subjective effects based on an interview 3 h after LSD.

TRD: treatment-resistant depression; DMT: *N,N*-dimethyltryptamine; SNRI: serotonin and noradrenaline reuptake inhibitor; SSRI: selective serotonin reuptake inhibitor; AD: antidepressant; SARI: serotonin antagonist and reuptake inhibitor; SMS: serotonin modulator and stimulator; AE: adverse event; MADRS: Montgomery Åsberg Depression Rating Scale; LSD: lysergic acid diethylamide; VAS: visual analogue scale; MAOI: monoamine oxidase inhibitor; TCA: tricyclic antidepressants; DTD: difficult-to-treat depression; MDD: Major Depressive Disorder; PTSD: post-traumatic stress disorder; : ADD: attention deficit disorder.

* Two weeks prior to second psilocybin dose, phenelzine and nortriptyline were stopped. Three days before second psilocybin dose, extended-release dextroamphetamine-amphetamine was initiated.

† Patient was advised to discontinue quetiapine for one week prior to first dose.

‡ Worsening of symptoms is hypothesized to be the result of moderate discontinuation symptoms after stopping the SNRI duloxetine (i.e. dizziness, muscle pain, and fatigue).

§ scores for Emotional Breakthrough Inventory were provided through correspondence with first author.

¶ Study population consisted primarily of MDD (50.8%) and anxiety (47.6%) but also included eating disorder (1.6%), OCD (3.2%), ADHD (23.8%), addiction (4.8%), personality disorder (3.2%), bipolar disorder (17.5%), hallucinogen persisting perception disorder (4.8%), alcohol addiction (1.6%), schizophrenia (1.6%), and psychotic disorder (1.6%). ||Reports primarily included psilocybin (*n* = 45, 71.4%) followed by LSD (*n* = 13, 20.6%), DMT/5-MeO-DMT (*n* = 3, 4.8%), and *ayahuasca* (*n* = 2, 3.2%).

** Data of “psychological,” “autonomic,” and “neurological” assessments are not reported in the article.

*** Subjects also received the antihypertensive reserpine (2 mg).

## Results

### Study flow

The search yielded 483 records, and 479 records remained after the removal of duplicates and the addition of records through reference list searching (Figure 1). We screened 479 titles and abstracts, which resulted in 21 articles assessed for eligibility. Two full-text articles were excluded because one did not evaluate the concomitant use of ADs and the other assessed the impact of pre-treatment with reserpine, which is not a conventional AD. Ultimately, 19 studies were included in this scoping review. Five studies were published in the 1960s and four studies published in the 1990s, whereas the remaining 10 studies were published after 2022.

### Safety

A total of 13 studies evaluated the safety of the concomitant use of ADs and psychedelics (Becker et al., 2022, 2025; Barnett et al., 2024; Callaway and Grob, 1998; DeMaar et al., 1960; Do et al., 2024; Falchi-Carvalho et al., 2025; Goodwin et al., 2023; Grof and Dytrych, 1965; Gukasyan et al., 2023; Resnick et al., 1964; Sakai et al., 2024; Vojtĕchovský et al., 1968), two randomized, double-blind placebo-controlled crossover trials (Becker et al., 2022, 2025), one double-blind placebo-controlled trial (DeMaar et al., 1960), one placebo-controlled trial (Vojtĕchovský et al., 1968), three case reports (Do et al., 2024; Barnett et al., 2024; Callaway and Grob, 1998), one retrospective survey (Gukasyan et al., 2023), and one qualitative study (Sakai et al., 2024).

Furthermore, the study populations taking ADs and psychedelics concomitantly within these studies consisted primarily of the general population (*n* = 1044) (Gukasyan et al., 2023; Sakai et al., 2024) followed by healthy volunteers (*n* = 66) (Becker et al., 2022, 2025; DeMaar et al., 1960; Resnick et al., 1964), treatment-resistant depression (TRD) (*n* = 34) (Barnett et al., 2024; Goodwin et al., 2023; Falchi-Carvalho et al., 2025), “neurotic” patients (*n* = 14) (Grof and Dytrych, 1965), abstinent alcoholics (*n* = 9) (Vojtĕchovský et al., 1968), difficult-to-treat depression (*n* = 1) (Do et al., 2024), and mild MDD (*n* = 1) (Callaway and Grob, 1998).

A total of seven studies looked at psilocybin (Barnett et al., 2024; Becker et al., 2022; Do et al., 2024; Goodwin et al., 2023; Gukasyan et al., 2023; Sakai et al., 2024; Vojtĕchovský et al., 1968). Although two studies did not report on the dosage of psilocybin (Gukasyan et al., 2023; Sakai et al., 2024), three studies looked at 25 mg (Becker et al., 2022; Do et al., 2024; Goodwin et al., 2023), and only one study looked at 20 mg (Vojtĕchovský et al., 1968). One study also included a batch of dried unknown psilocybin-containing mushrooms (3 g) and another batch of dried *psilocybe cubensis* (1 g) mushrooms (Barnett et al., 2024). There were four studies that evaluated LSD (Becker et al., 2025; DeMaar et al., 1960; Grof and Dytrych, 1965; Resnick et al., 1964) with dosages varying from 40 to 75 mcg (Resnick et al., 1964), 25–50–100 mcg (DeMaar et al., 1960), 100 mcg (Becker et al., 2025), and 150–500 mcg (Grof and Dytrych, 1965). Only one study looked at two doses of DMT (15 and 60 mg) (Falchi-Carvalho et al., 2025) and another at the DMT-containing brew *ayahuasca*, where the dosage was 100 ml (Callaway and Grob, 1998).

Only three studies included psychological support during psychedelic treatment, such as preparation, dosing supervision, and integration (Do et al., 2024; Falchi-Carvalho et al., 2025; Goodwin et al., 2023). Two studies explicitly reported features of setting (e.g. paintings and dimmed lights) (Falchi-Carvalho et al., 2025; Goodwin et al., 2023), whereas three studies were in (“calm”) hospital rooms (Becker et al., 2022, 2025; DeMaar et al., 1960), and four studies in uncontrolled, naturalistic settings (e.g. psychedelic retreat or ceremony) (Barnett et al., 2024; Callaway and Grob, 1998; Gukasyan et al., 2023; Sakai et al., 2024).

A total of four studies included a range of ADs (Barnett et al., 2024; Do et al., 2024; Falchi-Carvalho et al., 2025; Gukasyan et al., 2023), such as SSRIs, SNRIs, TCAs, MAOIs, and/or atypical ADs (e.g. vortioxetine). Five studies specifically assessed SSRIs (Becker et al., 2022, 2025; Callaway and Grob, 1998; Goodwin et al., 2023; Sakai et al., 2024) and four studies did so with MAOIs (DeMaar et al., 1960; Grof and Dytrych, 1965; Resnick et al., 1964; Vojtĕchovský et al., 1968). There were eight studies reporting on the length of AD treatment prior to psychedelic administration (Barnett et al., 2024; Becker et al., 2022, 2025; Callaway and Grob, 1998; Goodwin et al., 2023; Grof and Dytrych, 1965; Gukasyan et al., 2023; Resnick et al., 1964; Vojtĕchovský et al., 1968), which varied from 2 days (Vojtĕchovský et al., 1968), 2–5 weeks (Barnett et al., 2024; Becker et al., 2022; Grof and Dytrych, 1965; Resnick et al., 1964), 6 weeks (Becker et al., 2025), “several” months (Callaway and Grob, 1998), or up to more than 1 year (Goodwin et al., 2023; Gukasyan et al., 2023). Although three studies did not report the length of AD treatment (DeMaar et al., 1960; Do et al., 2024; Sakai et al., 2024), there was one study that did so only for the second dose of *psilocybe cubensis* (i.e. 2 weeks) (Barnett et al., 2024). Finally, there were three studies that included other concomitant psychotropic medications (Barnett et al., 2024; Do et al., 2024; Falchi-Carvalho et al., 2025).

Ten studies evaluating the safety of the concomitant use of ADs and classic psychedelics showed no signs of serotonin toxicity or syndrome (Barnett et al., 2024; Becker et al., 2022, 2025; DeMaar et al., 1960; Do et al., 2024; Falchi-Carvalho et al., 2025; Goodwin et al., 2023; Grof and Dytrych, 1965; Resnick et al., 1964; Vojtĕchovský et al., 1968). This was observed in psilocybin studies with SSRIs (Becker et al., 2022; Goodwin et al., 2023; Gukasyan et al., 2023; Sakai et al., 2024), SNRIs (Gukasyan et al., 2023), MAOIs (Barnett et al., 2024; Vojtĕchovský et al., 1968), TCAs (Barnett et al., 2024), SNRIs (Do et al., 2024; Gukasyan et al., 2023), and/or atypical AD (Do et al., 2024; Gukasyan et al., 2023), as well as LSD studies with an SSRI (Becker et al., 2025) and MAOIs (DeMaar et al., 1960; Grof and Dytrych, 1965; Resnick et al., 1964). Two ascending doses of DMT (15 and 60 mg) on the same day together with the concomitant use of either an SSRI, SNRI, serotonin antagonist and reuptake inhibitor (SARI), serotonin modulator and stimulators, or atypical AD (e.g. bupropion) did not result in serotonin toxicity or syndrome (Falchi-Carvalho et al., 2025). However, one qualitative study showed that two out of 433 reports were suggestive of severe serotonin syndrome (e.g. seizure and muscle rigidity) (Sakai et al., 2024). In addition, one retrospective survey indicated that 2.8% (*n* = 55) believed they had developed serotonin syndrome, but the study authors were unable to confirm due to the absence of hospital visit reports or formal diagnoses (Gukasyan et al., 2023). Finally, one case study involving a 36-year old man who received the SSRI fluoxetine 20 mg daily for several months for his mild MDD developed symptoms of serotonin syndrome (Boyer and Shannon, 2005), approximately one hour after 100 ml of *ayahuasca* (i.e. gross motor tremors, sweating, shivering, severe nausea, vomiting, as well as disorientation and confusion) but without any long-term adverse sequelae (Callaway and Grob, 1998). This was probably precipitated by MAOIs present within *ayahuasca*, which then resulted in dangerous levels of intrasynaptic serotonin (Malcolm and Thomas, 2022).

All 13 included studies reported on (serious) adverse events (AEs) during the concomitant use of ADs and classic psychedelics (Barnett et al., 2024; Becker et al., 2022, 2025; Callaway and Grob, 1998; DeMaar et al., 1960; Do et al., 2024; Falchi-Carvalho et al., 2025; Goodwin et al., 2023; Grof and Dytrych, 1965; Gukasyan et al., 2023; Resnick et al., 1964; Sakai et al., 2024; Vojtĕchovský et al., 1968), which primarily included headaches (Barnett et al., 2024; Becker et al., 2022, 2025; Do et al., 2024; Falchi-Carvalho et al., 2025; Goodwin et al., 2023; Sakai et al., 2024), nausea (Barnett et al., 2024; Becker et al., 2022, 2025; Callaway and Grob, 1998; Sakai et al., 2024), and increases in blood pressure (Barnett et al., 2024; Becker et al., 2025; Do et al., 2024; Falchi-Carvalho et al., 2025; Goodwin et al., 2023; Vojtĕchovský et al., 1968) . Other less frequent AEs consisted of pharyngeal discomfort and coughing (Falchi-Carvalho et al., 2025), impaired concentration and feeling dull (Becker et al., 2025), vomiting (Callaway and Grob, 1998; Sakai et al., 2024), pupil dilation (Vojtĕchovský et al., 1968), abdominal pain (Barnett et al., 2024), chest pain (Barnett et al., 2024), chest heaviness (Goodwin et al., 2023), heart palpitations (Barnett et al., 2024), and increased heart rate (Do et al., 2024; Falchi-Carvalho et al., 2025). Psychological AEs were paranoia, memory loss, or confusion (Sakai et al., 2024), distress (Barnett et al., 2024), and “massive psychotic symptomatology” (Grof and Dytrych, 1965). Furthermore, one retrospective survey showed that only 4% of their entire sample (*n* = 611) indicated an AE (Gukasyan et al., 2023), but it was not specified.

Notably, three studies found that the concomitant use of ADs was associated with a better safety profile compared to placebo, illustrated by (significant) decreases in systolic and diastolic blood pressure (Becker et al., 2022; DeMaar et al., 1960), heart rate (Becker et al., 2025), pupil dilation (Becker et al., 2022; DeMaar et al., 1960), mean arterial pressure (Becker et al., 2022), pupil dilation (Becker et al., 2022), and overall (sub)acute AEs (Becker et al., 2025). In addition, three studies found no significant differences compared to placebo in terms of blood pressure (Becker et al., 2025), mean arterial pressure (Becker et al., 2025), rate pressure product (Becker et al., 2025), body temperature (Becker et al., 2025), QTc interval (Becker et al., 2022, 2025), and/or other (sub)acute AEs (e.g. headaches, nausea, flashbacks, impaired concentration, feeling of weakness, abdominal bloating, lack of energy, insomnia, dry mouth, thirst) (Becker et al., 2022, 2025; Vojtĕchovský et al., 1968). One study found no clinically meaningful changes in suicidal ideation, clinical laboratory tests, ECG, and QTc interval from baseline to 3-week follow-up (Goodwin et al., 2023). Finally, one case study documented a hypertensive emergency and ST-elevation myocardial infarction (STEMI) in a patient following ingestion of dried *psilocybe cubensis* mushrooms after a two-week pretreatment period with the MAOI tranylcypromine and extended-release dextroamphetamine-amphetamine, suggesting a possible interaction between both medications and the phenylethylamine found within *psilocybe* mushrooms (Barnett et al., 2024).

### Treatment efficacy

Seven studies evaluated treatment efficacy following the concomitant use of ADs and psychedelics(Barnett et al., 2024; Barbut Siva et al., 2024; Callaway and Grob, 1998; Do et al., 2024; Falchi-Carvalho et al., 2025; Goodwin et al., 2023; Rosenblat et al., 2023). This included four case studies (Barnett et al., 2024; Callaway and Grob, 1998; Do et al., 2024; Rosenblat et al., 2023), two open-label trials (Falchi-Carvalho et al., 2025; Goodwin et al., 2023), and one prospective survey (Barbut Siva et al., 2024).

The study populations varied from patients with treatment-resistant depression (TRD) (*n* = 35) (Barnett et al., 2024; Goodwin et al., 2023; Rosenblat et al., 2023; Falchi-Carvalho et al., 2025), difficult-to-treat depression (*n* = 1) (Do et al., 2024), mild MDD (*n* = 1) (Callaway and Grob, 1998), to self-reported psychiatric diagnoses (*n* = 131) (Barbut Siva et al., 2024).

The majority of studies (*n* = 4) assessed psilocybin (Barnett et al., 2024; Do et al., 2024; Goodwin et al., 2023; Rosenblat et al., 2023), whereas one study looked at DMT (Falchi-Carvalho et al., 2025) and another at the DMT-containing brew *ayahuasca* (Callaway and Grob, 1998). One study evaluated a range of psychedelics, particularly psilocybin (71.4%), LSD (20.6%), *ayahuasca* (3.2%), and DMT/5-MeO-DMT (4.8%) (Barbut Siva et al., 2024). The dosage for psilocybin was 25 mg (Do et al., 2024; Goodwin et al., 2023; Rosenblat et al., 2023), 15 and 60 mg for DMT (Falchi-Carvalho et al., 2025), 100 ml for *ayahuasca* (Callaway and Grob, 1998), and was unknown in the study that assessed multiple psychedelic substances (Barbut Siva et al., 2024). Another study included one batch of dried unknown psilocybin-containing mushrooms (3 g) and one batch of dried *psilocybe cubensis* (1 g) mushrooms (Barnett et al., 2024).

There were four studies that included psychological support, such as preparation, dosing supervision, and integration (Do et al., 2024; Falchi-Carvalho et al., 2025; Goodwin et al., 2023; Rosenblat et al., 2023). Only two studies explicitly reported the setting in which a psychedelic was administered (Falchi-Carvalho et al., 2025; Goodwin et al., 2023), whereas three studies included an uncontrolled, naturalistic (Barnett et al., 2024; Barbut Siva et al., 2024; Callaway and Grob, 1998), and one study a “clinical” setting (Rosenblat et al., 2023).

One open-label trial (Goodwin et al., 2023) and one case study (Callaway and Grob, 1998) specifically evaluated SSRIs, whereas another case study did so with a SARI (Rosenblat et al., 2023). The remaining three studies included a range of ADs (Barbut Siva et al., 2024; Barnett et al., 2024; Do et al., 2024; Falchi-Carvalho et al., 2025), such as SSRIs, SNRIs, SARIs, TCAs, MAOIs, serotonin modulators and stimulators, and/or atypical ADs (e.g. vortioxetine). Only three studies included the length of AD treatment prior to psychedelic administration and varied from 2 weeks (Barnett et al., 2024), “several” months (Callaway and Grob, 1998), and up to ~14 months (Goodwin et al., 2023). Finally, there were three studies that included other concomitant psychotropic medications (Barnett et al., 2024; Do et al., 2024; Falchi-Carvalho et al., 2025). Four studies showed significant improvements in symptoms of depression following the concomitant use of ADs and classic psychedelics (Barbut Siva et al., 2024; Do et al., 2024; Falchi-Carvalho et al., 2025; Goodwin et al., 2023; Rosenblat et al., 2023). This was observed in psilocybin with SSRIs (Barbut Siva et al., 2024; Goodwin et al., 2023), SARI (Rosenblat et al., 2023), SNRIs (Barbut Siva et al., 2024; Do et al., 2024), and/or atypical AD (Do et al., 2024), as well as in LSD with SSRIs and/or SNRIs (Barbut Siva et al., 2024). Moreover, one study with DMT showed that decreases in depressive symptoms were greater in TRD patients with ADs compared to TRD patients without ADs, although this difference was only marginally significant (Falchi-Carvalho et al., 2025). Notably, three of studies showed full remission of depression following psilocybin (25 mg) (Goodwin et al., 2023; Rosenblat et al., 2023) and DMT (Falchi-Carvalho et al., 2025), despite the concomitant use of ADs. This effect was sustained at the 6-month follow-up in one case study with psilocybin (Rosenblat et al., 2023). Other (significant) improvements were observed in four studies in terms of “mental health” (Barnett et al., 2024), anxiety (Do et al., 2024), suicidality (Do et al., 2024; Falchi-Carvalho et al., 2025), illness severity (Goodwin et al., 2023), and well-being (Barbut Siva et al., 2024). Furthermore, one case study exemplified that the experience with *ayahuasca* and the concomitant use of the SSRI fluoxetine was considered a “valuable experience” in helping a patient reconcile with his wife after being unfaithful to her, despite the aforementioned symptoms of serotonin syndrome (Callaway and Grob, 1998).

Finally, one case study indicated a worsening in symptoms of depression, anxiety, and suicidality, which was hypothesized to be the result of discontinuing the SNRI duloxetine two weeks before a second dose of psilocybin (25 mg) (Do et al., 2024).

### Subjective effects

Nineteen studies evaluated the impact of concomitant AD use on the acute subjective effects of classic psychedelics (Barnett et al., 2024; Barbut Siva et al., 2024; Becker et al., 2022, 2025; Bonson, 1996; Bonson and Murphy, 1995; Callaway and Grob, 1998; DeMaar et al., 1960; Do et al., 2024; Falchi-Carvalho et al., 2025; Goodwin et al., 2023; Grof and Dytrych, 1965; Gukasyan et al., 2023; Resnick et al., 1964; Rosenblat et al., 2023; Sai-Halász, 1963; Sakai et al., 2024; Strassman, 1992; Vojtĕchovský et al., 1968).

The designs of these studies varied widely and consisted of two randomized double-blind placebo-controlled crossover trial (Becker et al., 2022, 2025), one double-blind placebo-controlled trial (DeMaar et al., 1960), one placebo-controlled trial (Vojtĕchovský et al., 1968), five open-label trials (Falchi-Carvalho et al., 2025; Goodwin et al., 2023; Grof and Dytrych, 1965; Resnick et al., 1964; Sai-Halász, 1963), five case studies (Barnett et al., 2024; Callaway and Grob, 1998; Do et al., 2024; Rosenblat et al., 2023; Strassman, 1992), one retrospective survey (Gukasyan et al., 2023), one prospective survey (Barbut Siva et al., 2024), and three qualitative studies (Bonson and Murphy, 1995; Bonson, 1996; Sakai et al., 2024) .

The study population was highly heterogeneous and varied from patients with MDD (*n* = 38) (Bonson, 1996; Bonson and Murphy, 1995), treatment-resistant depression (TRD) (*n* = 35) (Barnett et al., 2024; Goodwin et al., 2023; Rosenblat et al., 2023; Falchi-Carvalho et al., 2025), difficult-to-treat depression (*n* = 1) (Do et al., 2024), mild MDD (*n* = 1) (Callaway and Grob, 1998), anxiety disorder (*n* = 1) (Bonson, 1996), obsessionality (*n* = 1) (Strassman, 1992), dysthymia (*n* = 2) (Bonson, 1996; Strassman, 1992), post-traumatic stress disorder (*n* = 1) (Bonson, 1996), attention-deficit disorder (*n* = 1) (Bonson, 1996), “stress” (*n* = 1) (Bonson, 1996), being “neurotic” (*n* = 14) (Grof and Dytrych, 1965), self-reported psychiatric diagnoses (*n* = 131) (Barbut Siva et al., 2024), and abstinent alcoholics (*n* = 9) (Vojtĕchovský et al., 1968). Four studies looked at healthy volunteers (*n* = 73) (Becker et al., 2022; Resnick et al., 1964; Sai-Halász, 1963; DeMaar et al., 1960; Becker et al., 2025) and two studies at the general population (*n* = 1,044) (Sakai et al., 2024; Gukasyan et al., 2023).

There were eight studies that specifically assessed psilocybin (Barnett et al., 2024; Becker et al., 2022; Do et al., 2024; Goodwin et al., 2023; Gukasyan et al., 2023; Rosenblat et al., 2023; Sakai et al., 2024; Vojtĕchovský et al., 1968) and seven studies did so for LSD (Becker et al., 2025; Bonson, 1996; Bonson and Murphy, 1995; DeMaar et al., 1960; Grof and Dytrych, 1965; Resnick et al., 1964; Strassman, 1992). In addition, two studies looked at DMT (Falchi-Carvalho et al., 2025; Sai-Halász, 1963) and another at the DMT-containing brew *ayahuasca* (Callaway and Grob, 1998). Finally, one study included a range of psychedelics, particularly psilocybin (71.4%), LSD (20.6%), *ayahuasca* (3.2%), and DMT/5-MeO-DMT (4.8%) (Barbut Siva et al., 2024). The dosage for psilocybin was 25 mg in four studies (Becker et al., 2022; Do et al., 2024; Goodwin et al., 2023; Rosenblat et al., 2023) and 20 mg in only one study (Vojtĕchovský et al., 1968). There was also one study that included one batch of dried unknown psilocybin-containing mushrooms (3 g) and one batch of dried *psilocybe cubensis* (1 g) mushrooms (Barnett et al., 2024). Regarding LSD, the dosage varied widely from 25 mcg (DeMaar et al., 1960), 40 mcg (Resnick et al., 1964), 50 mcg (DeMaar et al., 1960), 75 mcg (Bonson, 1996; Resnick et al., 1964), 100 mcg (Becker et al., 2025; DeMaar et al., 1960), 150 mcg (Grof and Dytrych, 1965), 400 mcg (Grof and Dytrych, 1965), to 500 mcg (Bonson, 1996; Grof and Dytrych, 1965). The dosage for *ayahuasca* was 100 ml (Callaway and Grob, 1998) and for DMT it was 15 mg and 60 mg (Falchi-Carvalho et al., 2025) and 0.35–0.55 mg/kg and 0.65–0.84 mg/kg (Sai-Halász, 1963). There were five studies that did not report the specific dosage for any classic psychedelic (Barbut Siva et al., 2024; Bonson and Murphy, 1995; Gukasyan et al., 2023; Sakai et al., 2024; Strassman, 1992) .

There were four studies that included psychological support, such as preparation, dosing supervision, and integration (Do et al., 2024; Falchi-Carvalho et al., 2025; Goodwin et al., 2023; Rosenblat et al., 2023). The setting in which a psychedelic was administered was reported by only two studies, such as the use of dimmed lights and paintings in the room (Falchi-Carvalho et al., 2025; Goodwin et al., 2023). Finally, three studies featured a (calm) hospital room (Becker et al., 2025; Becker et al., 2022; DeMaar et al., 1960) and one study a “clinical” setting (Rosenblat et al., 2023). The remaining seven studies comprised uncontrolled, naturalistic settings (e.g. psychedelic retreat or ceremony) (Barbut Siva et al., 2024; Barnett et al., 2024; Bonson, 1996; Bonson and Murphy, 1995; Callaway and Grob, 1998; Gukasyan et al., 2023; Sakai et al., 2024).

Regarding AD class, seven studies evaluated a range of ADs (Barnett et al., 2024; Barbut Siva et al., 2024; Bonson, 1996; Bonson and Murphy, 1995; Do et al., 2024; Falchi-Carvalho et al., 2025; Gukasyan et al., 2023), including SSRIs, SNRIs, SARIs, TCAs, MAOIs, serotonin modulator and stimulators, and/or atypical ADs (e.g. bupropion or vortioxetine). Furthermore, six studies specifically assessed SSRIs (Becker et al., 2022, 2025; Callaway & Grob, 1998; Goodwin et al., 2023; Sakai et al., 2024; Strassman, 1992) and another five studies looked at MAOIs (DeMaar et al., 1960; Grof and Dytrych, 1965; Resnick et al., 1964; Sai-Halász, 1963; Vojtĕchovský et al., 1968). There was only one study that evaluated a SARI (Rosenblat et al., 2023). The length of AD treatment varied widely in 12 studies and ranged from 2 days (Vojtĕchovský et al., 1968), 4 days (Sai-Halász, 1963), 2 weeks (Barnett et al., 2024; Becker et al., 2022; Resnick et al., 1964), ~3 weeks (Bonson, 1996; Bonson and Murphy, 1995; Grof and Dytrych, 1965), 5 weeks (Resnick et al., 1964), 6 weeks (Becker et al., 2025), “several” weeks (Grof and Dytrych, 1965), less than 1 month (Gukasyan et al., 2023), “several” months (Callaway and Grob, 1998), ~14 months (Goodwin et al., 2023), more than 1 year (Gukasyan et al., 2023), and up to 3 years (Bonson, 1996; Bonson and Murphy, 1995). One study only reported the length of AD treatment for the second dose of *psilocybe cubensis* and was 2 weeks (Barnett et al., 2024). Finally, there were three studies that included other concomitant psychotropic medications (e.g. zolpidem) (Barnett et al., 2024; Do et al., 2024; Falchi-Carvalho et al., 2025).

Ten studies showed that the concomitant use of ADs (significantly) decreased the acute subjective effects of classic psychedelics (Barbut Siva et al., 2024; Becker et al., 2022; Bonson, 1996; Bonson & Murphy, 1995; Grof & Dytrych, 1965; Gukasyan et al., 2023; Resnick et al., 1964; Sai-Halász, 1963; Sakai et al., 2024; Strassman, 1992). Specifically, this relationship was observed in four psilocybin studies with SSRIs (Barbut Siva et al., 2024; Becker et al., 2022; Gukasyan et al., 2023; Sakai et al., 2024), SNRIs (Barbut Siva et al., 2024; Gukasyan et al., 2023), and/or atypical ADs (Gukasyan et al., 2023), six LSD studies with SSRIs (Barbut Siva et al., 2024; Bonson, 1996; Strassman, 1992), SNRIs (Barbut Siva et al., 2024), SARIs (Bonson, 1996), and/or MAOIs (Bonson and Murphy, 1995; Grof and Dytrych, 1965; Resnick et al., 1964), and one DMT study with MAOIs (Sai-Halász, 1963).

Furthermore, six studies illustrated that the concomitant use of ADs completely eliminated the acute subjective effects of classic psychedelics (Barnett et al., 2024; Bonson, 1996; Grof and Dytrych, 1965; Resnick et al., 1964; Rosenblat et al., 2023; Sai-Halász, 1963). This was shown in two psilocybin studies with MAOIs (Barnett et al., 2024), TCAs (Barnett et al., 2024), and/or SARIs (Rosenblat et al., 2023), three LSD studies with SSRIs (Bonson, 1996), SARIs (Bonson, 1996), and/or MAOIs (Grof and Dytrych, 1965; Resnick et al., 1964), and one DMT study with MAOIs (Sai-Halász, 1963).

Simultaneously, eight studies show that the concomitant use of ADs does not result in (significant) changes in the acute subjective effects of classic psychedelics (Becker et al., 2025; Callaway and Grob, 1998; DeMaar et al., 1960; Falchi-Carvalho et al., 2025; Goodwin et al., 2023; Gukasyan et al., 2023; Sakai et al., 2024; Vojtĕchovský et al., 1968). This was observed in five psilocybin studies with SSRIs (Goodwin et al., 2023; Gukasyan et al., 2023; Sakai et al., 2024), SNRIs (Do et al., 2024; Gukasyan et al., 2023), MAOIs (Vojtĕchovský et al., 1968), and/or atypical ADs (Do et al., 2024; Gukasyan et al., 2023), two LSD studies involving an SSRI (Becker et al., 2025) or MAOI (DeMaar et al., 1960), one DMT study involving a range of ADs (Falchi-Carvalho et al., 2025), and one study involving an SSRI and the DMT-containing brew *ayahuasca* and (Callaway and Grob, 1998).

In addition, four studies indicate an increased intensity of acute subjective effects (Bonson, 1996; Bonson and Murphy, 1995; Gukasyan et al., 2023; Sakai et al., 2024). This was observed in two psilocybin studies concurrent with SSRIs (Gukasyan et al., 2023; Sakai et al., 2024), SNRIs (Gukasyan et al., 2023) and/or atypical ADs (Gukasyan et al., 2023), and two LSD studies concurrent with an SSRI (Bonson, 1996) and/or TCAs (Bonson and Murphy, 1995).

Finally, one retrospective survey study showed that respondents (*n* = 128) were not sure whether their concomitant use of ADs impacted the acute subjective effects of psilocybin (Gukasyan et al., 2023).

## Discussion

In this scoping review, we identified 19 studies evaluating the concomitant use of conventional ADs and classic psychedelics. The main findings indicate that this combination appears generally safe and tolerable with no robust evidence of increased risk for serotonin toxicity or syndrome. Furthermore, some studies reported significant improvements in depression and other symptoms with combined treatment. A final observation was the potential attenuation of acute subjective effects of psychedelics, although other studies did not show this. These findings appear to challenge the common practice of AD discontinuation before psychedelic treatment.

One of the most important clinical implications of this scoping review is that the concomitant use of ADs could significantly increase access to psychedelic treatment. For instance, SSRIs are the most frequently prescribed ADs for reducing depressive symptoms in patients with MDD (Dold et al., 2016; Luo et al., 2020). Currently, only four out of 100 registered psilocybin studies allow the use of SSRIs while 84 exclude the use of SSRIs (Sakai et al., 2024). Yet, findings from this scoping review suggest that this combination is not reliably associated with increased risk of serotonin toxicity (Becker et al., 2022, 2025; Goodwin et al., 2023; Gukasyan et al., 2023; Sakai et al., 2024). In addition, adverse events in this scoping review were either very low (Gukasyan et al., 2023; Sakai et al., 2024) or similar to placebo (Becker et al., 2022, 2025; Goodwin et al., 2023) in terms of intensity and frequency, and seem to correspond with recent psilocybin trials without concomitant AD medication (Yerubandi et al., 2024). Furthermore, combining SSRIs with psilocybin may offer a promising approach for patients with MDD who are hesitant to discontinue ADs (Becker et al., 2025; Goodwin et al., 2023), particularly due to concerns regarding relapse or withdrawal symptoms (Maund et al., 2019). These concerns are well-founded as AD discontinuation symptoms can increase the risk of MDD relapse by 40% (Kato et al., 2021) and affect approximately 56% of patients (Davies and Read, 2019). Typically, symptoms following the discontinuation of SSRIs appear within the first few days and can last for several weeks, ranging from physical manifestations (e.g. headaches, malaise, insomnia, and nausea) to psychological effects (e.g. anxiety/agitation, irritability, and difficulty concentrating) (Fava et al., 2015; Renoir, 2013). Notably, preliminary results indicate that AD discontinuation symptoms may negatively impact the efficacy of psilocybin (Do et al., 2024; Erritzoe et al., 2024), although most recent evidence does not support this relationship (Marwood et al., 2024).

This scoping review further revealed the potential of ADs to attenuate the acute subjective effects of psychedelics, which could impact the efficacy of psychedelics. In the past, some have argued that the acute subjective effects are essential for therapeutic benefit (Yaden and Griffiths, 2021), as it tend to be a significant predictor for positive therapeutic outcomes in several psychiatric disorders (Hovmand et al., 2023). Conversely, it is argued that psychedelics may also exert therapeutic effects in the absence of acute subjective effects and are instead mediated by neurobiological mechanisms (Olson, 2020, 2022). Within this scoping review, significant improvements in mental health or depressive symptoms without any acute subjective effects was observed in two case studies involving *psilocybe* mushrooms (species unknown) concomitant with an MAOI and TCA (Barnett et al., 2024), and psilocybin concomitant with a SARI (Rosenblat et al., 2023). This therapeutic response can be explained by increases in neuroplasticity and brain-derived neurotrophic factor (BDNF) as demonstrated in rodent research involving classic psychedelics (Cameron et al., 2023; Ly et al., 2018; Moliner et al., 2023; Vargas et al., 2023). Notably, two randomized double-blind placebo-controlled crossover trials in this scoping review show that the significant increase of BDNF following psilocybin (Becker et al., 2022) and LSD (Becker et al., 2025) is not significantly affected by concurrent use of SSRIs compared to placebo in healthy volunteers. However, one open-label trial (Goodwin et al., 2023) and another prospective survey (Barbut Siva et al., 2024) in this scoping review revealed significant improvements in depressive symptoms following psilocybin or LSD alongside acute psychedelic effects despite concurrent use of SSRIs and/or SNRIs. In short, regardless of whether the acute subjective effects are responsible for efficacy, findings from this scoping review indicate that the concomitant use of ADs does not appear to negatively impact psychedelic treatment outcomes. Taken together, these findings challenge the current practice of AD discontinuation before psychedelic treatment.

Findings from this scoping review must be interpreted with caution due to a paucity of randomized controlled trials, with current evidence largely derived from case reports (Barnett et al., 2024; Do et al., 2024; Rosenblat et al., 2023; Callaway & Grob, 1998; Strassman, 1992), qualitative studies (Bonson, 1996; Bonson and Murphy, 1995; Sakai et al., 2024), surveys (Barbut Siva et al., 2024; Gukasyan et al., 2023), and/or open-label studies lacking a control group (DeMaar et al., 1960; Goodwin et al., 2023; Grof and Dytrych, 1965; Resnick et al., 1964; Sai-Halász, 1963) with highly heterogenous study populations. Furthermore, this scoping review highlights several critical knowledge gaps and warrant further investigation. First and foremost, the complex interplay between specific dosages of ADs and classic psychedelics remains poorly understood, necessitating controlled studies to elucidate specific dose-response relationships and the distinct effects of particular AD classes. The urgent need for clearer guidance is further underscored by recent survey data, illustrating that medication interactions (e.g. ADs) with psychedelics was among the most commonly desired educational topics reported by psychiatrists (Barnett et al., 2022). Secondly, while psilocybin has been relatively well-studied, data for LSD is limited (Becker et al., 2025) and exists primarily for the concomitant use of MAOIs that is also very old (DeMaar et al., 1960; Grof and Dytrych, 1965; Resnick et al., 1964), albeit showing a good safety profile and no signs of serotonin syndrome. Data for DMT (Falchi-Carvalho et al., 2025; Sai-Halász, 1963), *ayahuasca* (Callaway and Grob, 1998), and 5-MeO-DMT (Barbut Siva et al., 2024) is extremely limited. Further investigation is crucial to determine the precise risk of serotonin syndrome within these other classic psychedelics, particularly with specific combinations like *ayahuasca* and SSRIs/MAOIs (Callaway and Grob, 1998; Malcolm and Thomas, 2022). Thirdly, robust randomized placebo-controlled trials are essential to establish the true therapeutic potential of combined treatment, focusing on different patient populations (e.g. MDD, TRD, or post-traumatic stress disorder), employing standardized clinical outcome measures, other psychotropic medications (e.g. antipsychotics), and including follow-up assessments to evaluate long-term safety and efficacy. Fourthly, the potential attenuation of acute psychedelic effects by different ADs, the duration of AD treatment, as well as the variability in individual experiences, demand further exploration through psychometric scales typically used within psychedelic research (de Deus Pontual et al., 2023; Hovmand et al., 2023; Yaden et al., 2024). Finally, it is unclear to what extent psychological support and setting affect combined treatment due to heterogeneous and limited data (Falchi-Carvalho et al., 2025; Goodwin et al., 2023; Rosenblat et al., 2023). Importantly, both components are implicated in safety and treatment efficacy (Johnson et al., 2008), and setting seems able to moderate the acute subjective effects of psychedelics (Hartogsohn, 2016; Olson et al., 2020). Future researchers should employ the Template for Intervention Description and Replication (Seybert et al., 2025) and Reporting of Setting in Psychedelic Clinical Trials (ReSPCT) guidelines (Pronovost-Morgan et al., 2025). Respectively, this allows them to exemplify the nature and extent of psychological support and setting and how it influences safety, tolerability, and efficacy of psychedelics. Future research addressing these knowledge gaps and recommendations could significantly enhance the rigor, replicability, and generalisability of findings, ensuring the safe and effective clinical implementation of psychedelics in general as well as combined treatment with ADs.

In conclusion, findings from this scoping review indicate that the discontinuation of ADs within psychedelic research may be unnecessary and unwarranted, given the very limited evidence that ADs increase the risk of serotonin syndrome or other adverse events. Furthermore, combined treatment does not appear to impact efficacy of psychedelics. While there is some evidence that concomitant ADs may attenuate the acute subjective effects of psychedelics, it does not appear to significantly decrease therapeutic outcomes. The continuation of ADs would significantly increase accessibility for psychedelic treatment. Future research is needed to disentangle the impact of the concomitant use of ADs and psychedelics and enhance the rigor, replicability, and generalisability of findings.

## Supplemental Material

sj-docx-1-jop-10.1177_02698811251368360 – Supplemental material for Concomitant use of antidepressants and classic psychedelics: A scoping review Supplemental material, sj-docx-1-jop-10.1177_02698811251368360 for Concomitant use of antidepressants and classic psychedelics: A scoping review by Stephan C. Tap, Kelan Thomas, Tomáš Páleníček, Dea S. Stenbæk, Albino J. Oliveira-Maia, Jens van Dalfsen and Robert Schoevers in Journal of Psychopharmacology
