# Supplementary material for: Concomitant use of antidepressants and classic psychedelics: A scoping review
Source: J Psychopharmacol. 2025 Sep 12;39(10):1072–88. doi: 10.1177/02698811251368360 (PMC12572353; doi:10.1177/02698811251368360)
Supplement: sj-docx-1-jop-10.1177_02698811251368360 – Supplemental material for Concomitant use of antidepressants and classic psychedelics: A scoping review [file sj-docx-1-jop-10.1177_02698811251368360.docx]

**Appendix A. Search strategy**

**PubMed (MEDLINE)**

("Antidepressive Agents"[Mesh] OR "Selective Serotonin Reuptake Inhibitors"[Mesh] OR “Antidepressive Agents, Tricyclic”[Mesh] OR “Antidepressive Agents, Second-Generation”[Mesh] OR “Monoamine Oxidase Inhibitors”[Mesh] OR antidepress*[tiab] OR "selective serotonin reuptake inhibitor*"[tiab] OR SSRI*[tiab] OR "serotonin norepinephrine reuptake inhibitor*"[tiab] OR SNRI*[tiab] OR "monoamine oxidase inhibitor*"[tiab] OR MAOI*[tiab] OR tricyclic antidepressant*[tiab] OR TCA[tiab] OR TCAs[tiab] OR citalopram[tiab] OR escitalopram[tiab] OR fluoxetine[tiab] OR fluvoxamine[tiab] OR paroxetine[tiab] OR sertraline[tiab] OR venlafaxine[tiab] OR duloxetine[tiab] OR bupropion[tiab] OR mirtazapine[tiab]) AND ("Hallucinogens"[Mesh] OR psychedelic*[tiab] OR hallucinogen*[tiab] OR entheogen*[tiab] OR "lysergic acid diethylamide"[tiab] OR LSD[tiab] OR psilocybin[tiab] OR N,N-dimethyltryptamine[tiab] OR N,N-DMT[tiab] OR DMT[tiab] OR 5-methoxy-N,N-dimethyltryptamine[tiab] OR 5-MeO-DMT[tiab] OR ayahuasca[tiab] OR mescaline[tiab] OR peyote[tiab]) AND ("Drug Interactions"[Mesh] OR "Drug Combinations"[Mesh] OR concomitant[tiab] OR impact[tiab] OR continuation*[tiab] OR interaction*[tiab] OR combination*[tiab] OR co-administ*[tiab] OR coadminist*[tiab] OR "concurrent use"[tiab] OR polysubstance[tiab] OR polydrug[tiab] OR "combined use"[tiab] OR "combined administration"[tiab] OR "simultaneous use"[tiab]) NOT (rodent*[tiab] OR rat[tiab] OR rats[tiab] OR mouse[tiab] OR mice[tiab] OR animals[tiab])

**Embase**

('Antidepressive Agents' OR 'Serotonin Uptake Inhibitors' OR antidepress*:ti,ab OR 'selective serotonin reuptake inhibitor*':ti,ab OR SSRI*:ti,ab OR 'serotonin norepinephrine reuptake inhibitor*':ti,ab OR SNRI*:ti,ab OR 'monoamine oxidase inhibitor*':ti,ab OR MAOI*:ti,ab OR 'tricyclic antidepressant*':ti,ab OR TCA*:ti,ab OR citalopram:ti,ab OR escitalopram:ti,ab OR fluoxetine:ti,ab OR fluvoxamine:ti,ab OR paroxetine:ti,ab OR sertraline:ti,ab OR venlafaxine:ti,ab OR duloxetine:ti,ab OR bupropion:ti,ab OR mirtazapine:ti,ab) AND (Hallucinogens OR N,N-Dimethyltryptamine OR Psilocybine OR psychedelic*:ti,ab OR hallucinogen*:ti,ab OR entheogen*:ti,ab OR 'lysergic acid diethylamide':ti,ab OR LSD:ti,ab OR psilocybin*:ti,ab OR DMT:ti,ab OR dimethyltryptamine:ti,ab OR ayahuasca:ti,ab OR mescaline:ti,ab OR peyote:ti,ab OR 5-MeO-DMT:ti,ab OR N,N-DMT:ti,ab) AND ('Drug Interactions' OR 'Drug Combinations' OR concomitant:ti,ab OR impact:ti,ab OR continuation*:ti,ab OR interaction*:ti,ab OR combination*:ti,ab OR co-administ*:ti,ab OR coadminist*:ti,ab OR 'concurrent use':ti,ab OR polysubstance:ti,ab OR polydrug:ti,ab OR 'combined use':ti,ab OR 'combined administration':ti,ab OR 'simultaneous use':ti,ab) NOT (rat*:ti,ab OR mouse:ti,ab OR mice:ti,ab OR animals:ti,ab)

**Scopus**

( TITLE-ABS-KEY("antidepressive agent*" OR "selective serotonin reuptake inhibitor*" OR SSRI* OR "serotonin norepinephrine reuptake inhibitor*" OR SNRI* OR "monoamine oxidase inhibitor*" OR MAOI* OR "tricyclic antidepressant*" OR TCA OR TCAs OR citalopram OR escitalopram OR fluoxetine OR fluvoxamine OR paroxetine OR sertraline OR venlafaxine OR duloxetine OR bupropion OR mirtazapine) ) AND ( TITLE-ABS-KEY(psychedelic* OR hallucinogen* OR entheogen* OR "lysergic acid diethylamide" OR LSD OR psilocybin OR "N,N-dimethyltryptamine" OR N,N-DMT OR DMT OR "5-methoxy-N,N-dimethyltryptamine" OR "5-MeO-DMT" OR ayahuasca OR mescaline OR peyote) ) AND ( TITLE-ABS-KEY("drug interaction*" OR "drug combination*" OR concomitant OR impact OR continuation* OR interaction* OR combination* OR co-administ* OR coadminist* OR "concurrent use" OR polysubstance OR polydrug OR "combined use" OR "combined administration" OR "simultaneous use") ) AND NOT ( TITLE-ABS-KEY(rodent* OR rat OR rats OR mouse OR mice OR animal*) )
